# A bibliometric analysis from 2013 to 2024 reveals research hotspots and trends in the connection between atrial fibrillation and cryoballoon: An observational study

**DOI:** 10.1097/MD.0000000000038435

**Published:** 2024-06-14

**Authors:** Jing Lu, Nan Zhang, Fan Yang, Yu Gao, Yan Ren, Dengfeng Ma

**Affiliations:** a Department of Cardiology, Taiyuan Central Hospital of Shanxi Medical University, Taiyuan, Shanxi, China.

**Keywords:** atrial fibrillation, bibliometric analysis, Citespace, cryoballoon, knowledge mapping

## Abstract

Using bibliometric analysis, this study attempted to provide an overview of the current state of research and key findings regarding the relationship between atrial fibrillation (AF) and cryoballoons in general. We gathered the literature from the Web of Science (WOS) database covering the last 11 years (2013–2024) pertaining to AF and cryoballoons, and we used Citespace to evaluate the mapping of knowledge. The findings demonstrated that there were 1986 articles concerning AF and cryoballoons, with a faster growth after 2018. The United States, Vrije Universiteit Brussel, and Chierchia, Gian-Battista are the nation, organization, and writer with the highest number of publications. Kuck KH (2016) is the most frequently mentioned reference as well (488). We know that Vrije Universiteit Brusse in the Belgium has emerged as 1 of this discipline’s leading research forces based on a 10-year bibliometric investigation. Prominent universities and developed nations form the finest alliances for research on cryoballoons and AF.

## 1. Introduction

With a prevalence of 1%–2% in the general population, atrial fibrillation (AF) is the most common persistent arrhythmia seen in clinical practice. Its prevalence rises dramatically with age, from 1.0% at age 50 to 4% at age 65 and 12% at age 80. AF is associated with significant impairments in functional capacity and quality of life, though it is rarely acutely life-threatening. The degree of impairment is comparable to or worse than in patients with heart failure or coronary disease, and the illness intrusiveness metrics are comparable to those of chronic hemodialysis.^[1,2]^ Uncontrolled AF is independently linked to a 5-fold increase in thromboembolism risk and a 4-fold increase in death risk.^[3]^

Cryoballoon catheter ablation produced clinically meaningful improvements in patient-reported outcomes (e.g., symptoms and quality of life) and significantly improved arrhythmia outcomes in patients with treatment-naive AF when compared to initial antiarrhythmic drug therapy (Central Illustration). It also significantly reduced the risk of adverse events.These results are pertinent to educating patients, healthcare professionals, and systems about the first therapeutic option for individuals with treatment-naive AF, which is rhythm-control therapy.^[4]^

Cryoballoon research is evolving so fast that it is difficult to fully comprehend its current state and hotspots. Bibliometrics is the quantitative analysis of certain subjects using a variety of databases, including Pubmed and Web of Science (WOS), among others.^[5,6]^ Among these, the Citespace software enables WOS to carry out quantitative scientific analysis. Among these, the Citespace software enables WOS to carry out quantitative scientific analysis.^[7,8]^ By using bibliometrics, readers may fully comprehend the frontiers, trends, and hotspots in this field.^[5,7]^ It looks at the turning points in the evolution of several topic areas. Bibliometrics includes co-author, co-citation, and co-occurrence analysis.^[8–11]^

Since the topic of cryoballoons is still very complex and constantly evolving, sometimes even contradicting itself, research findings are widely published in scientific articles.^[12]^ On the one hand, AF and cryoballoon collaborations have advanced significantly due to the rise in published research. However, because there are so many dispersed, occasionally redundant studies in this field, it is still challenging to obtain a comprehensive understanding of the state of research on cryoballoon and AF today. There isn’t yet a bibliometric method for researching the connection between AF and cryoballoons. To this end, Citespace was used to investigate the trends and worldwide significance of AF and cryoballoons in the WOS database from January 1, 2013, to March 26, 2024.

## 2. Method

### 2.1. Source of literature

We enter the subject terms into the WOS database (SCI, SSCI, ESCI, etc): TS= ((Atrial Fibrillations) OR (Auricular Fibrillation) OR (Auricular Fibrillations) OR (Persistent Atrial Fibrillation) OR (Persistent Atrial Fibrillations) OR (Familial Atrial Fibrillation) OR (Familial Atrial Fibrillations) OR (Fibrillation, Familial Atrial) OR (Paroxysmal Atrial Fibrillation) OR (Paroxysmal Atrial Fibrillations)) AND (Cryoballoon). The search scope of the database is from January 1, 2013, to March 30, 2024, and the language type was English. Through the literature search (articles, reviews, meeting abstracts, etc), 1986 records were obtained. The WOS database comes from the Taiyuan Central Hospital of Shanxi Medical University. All the articles containing “search terms” have been checked (because some retrieved articles may not necessarily be 100% relevant). Two researchers worked independently to screen the literature and assess its quality. These publications provided bibliometric information, such as the publication date, the yearly quantity of publications/citations, the countries/regions, the institutions, the authors, the journals, the references, and the keywords.

### 2.2. Analysis software

Citespace analysis software version is Version 5.6. R2.^[12]^

### 2.3. Download and import of data

The results of the retrieved subject terms were exported, and the file format was kept as “plain text.”

### 2.4. Parameter setting

Time slicing (from 2013 to 2024); node type (checked individually); 50 selection criteria; trimming (pathfinder); and visualization (showing the merged network, cluster view-static).

### 2.5. Statistical methods

**Figure 1.**
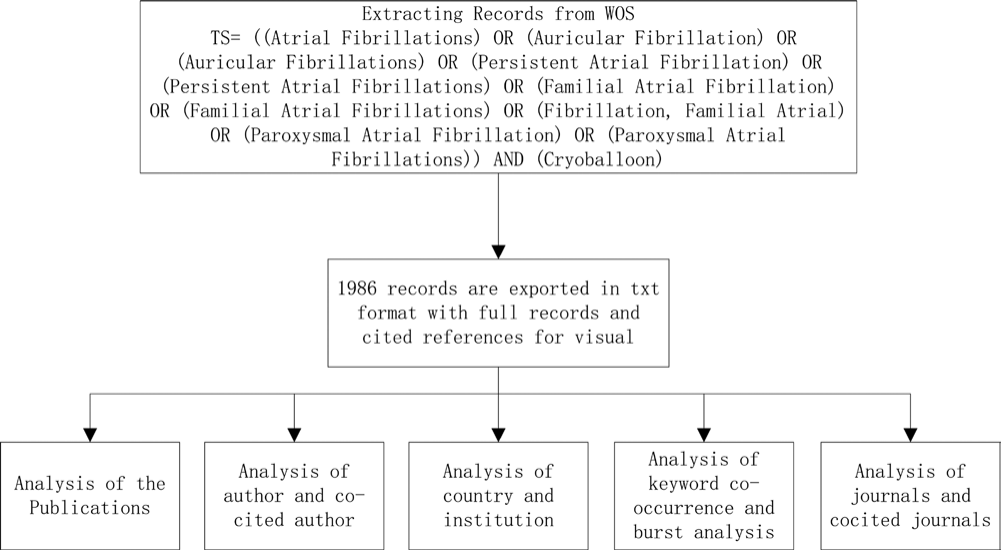
Analysis flow chart of atrial fibrillation and cryoballoon.

Microsoft Office Excel 2021 was used to manage data and analyze annual publications. All literature has been scientifically analyzed. Some data obtained include core countries, institutions, authors, co-cited references, keyword co-occurrence.^[13–15]^ The detailed analysis flow is shown in Figure 1.

## 3. Results

### 3.1. Analysis of the publications

**Figure 2.**
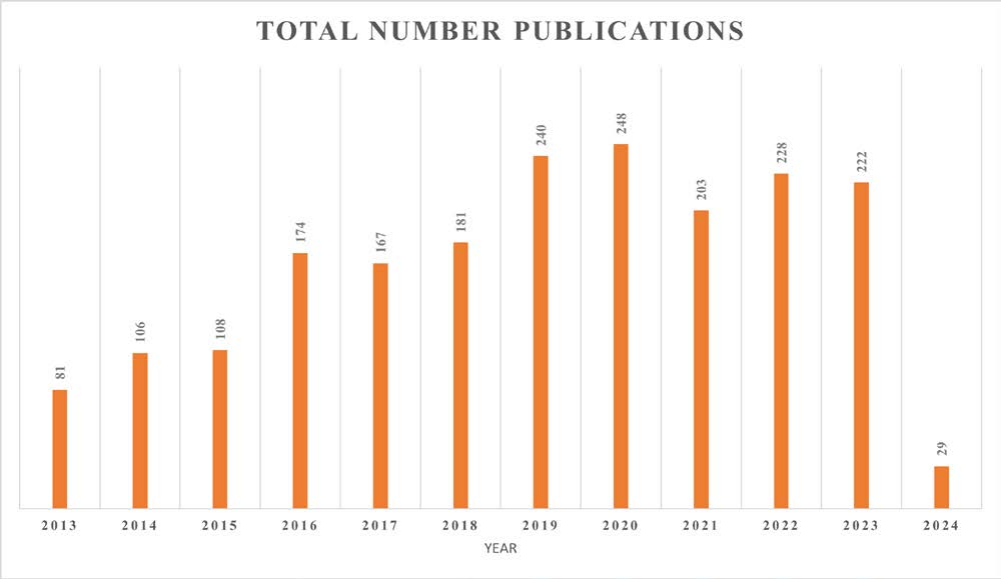
The number of atrial fibrillation and cryoballoon publications indexed by WOS from 2013 to 2024.

The overall number of papers fluctuated during the research period. The research is broken down into 2 stages, as shown in Figure 2: the first stage spans the years 2013 through 2015, and the second stage spans the years 2016–2023. There was a surge in development during the second stage. In 2016, there were 174 references published in the magazine; by 2020, there were 248 references. These results imply that during the past 8 years, AF and cryoballoon research have become more important.

### 3.2. Analysis of countries and institutions

**Figure 3.**
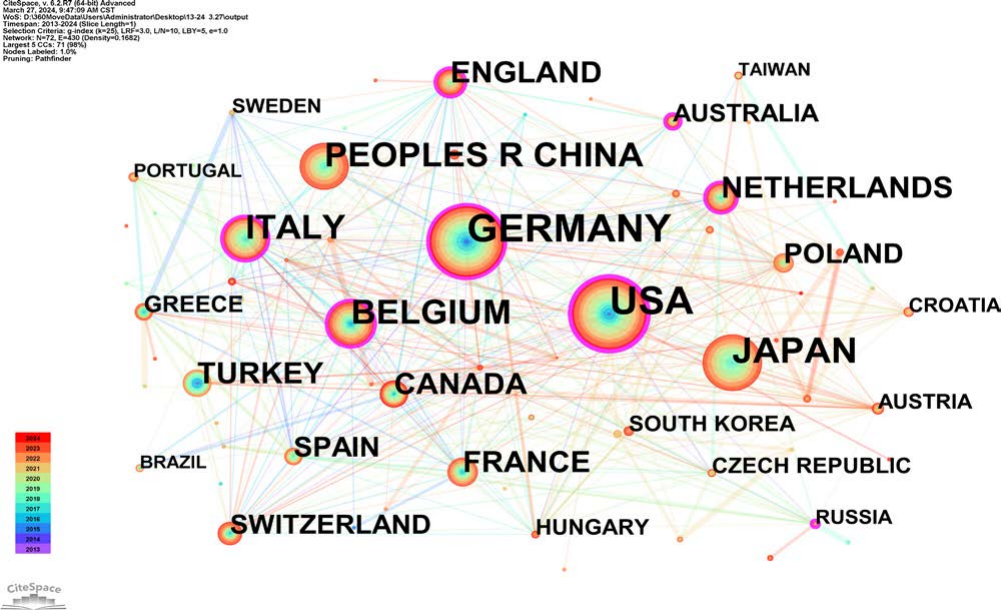
Analysis of the country map from 2013 to 2024.

A country map was generated (Fig. 3). About 72 countries published 2773 references. The USA, GERMANY, JAPAN, PEOPLES R CHINA, and BELGIUM are the top 5 countries (Table 1). The USA (0.34), ENGLAND (0.2), GERMANY (0.13), BELGIUM (0.13), and ITALY (0.13) are the top 5 countries from centrality (purple round). An analysis of publications and centrality shows that the USA, GERMANY, and BELGIUM were the main research forces in the study of the relationship between AF and cryoballoon. USA, PEOPLES R CHINA, and GERMANY have been increasingly interested in this field.

**Table 1:** Top 5 countries and institutions researching atrial fibrillation and cryoballoon.

Ranking	Country	Publications	Ranking	Institution	Publications
1	USA	415	1	Vrije Universiteit Brussel	121
2	GERMANY	407	2	University Hospital Brussels	98
3	JAPAN	276	3	Asklepios Klinik St. Georg	97
4	PEOPLES R CHINA	205	4	Tokyo Medical & Dental University (TMDU)	71
5	BELGIUM	195	5	Maria Cecilia Hospital	62

**Figure 4.**
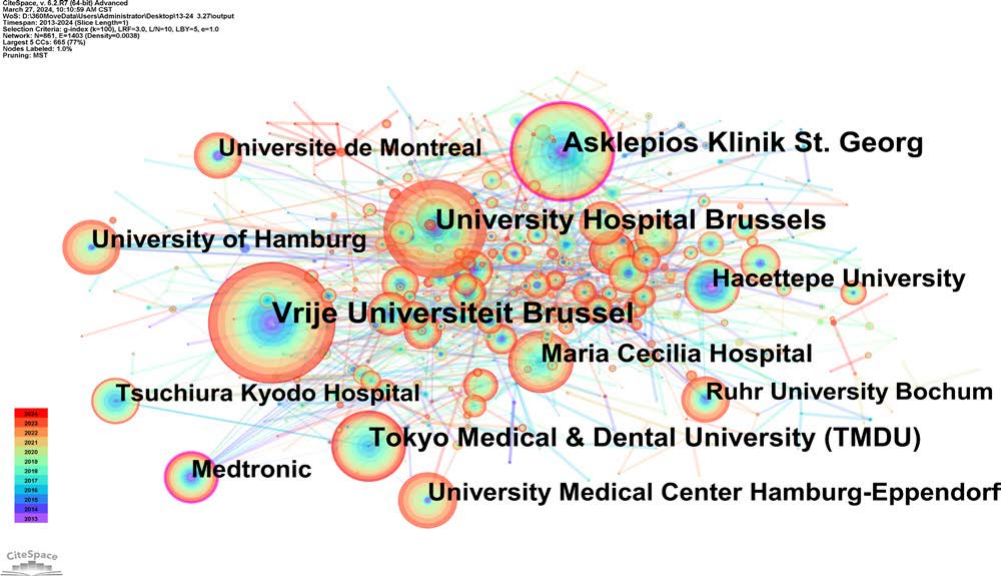
Institutional map researching atrial fibrillation and cryoballoon from 2013 to 2024.

Generated an institution map with 861 nodes and 1403 links (Fig. 4). The 4811 publications have been published in 861 institutions. The Vrije Universiteit Brussel, University Hospital Brussels, Asklepios Klinik St. Georg, Tokyo Medical & Dental University (TMDU), and Maria Cecilia Hospital are the top 5 institutions (Table 1). Regarding centrality, the top 3 institutions were Asklepios Klinik St. Georg (0.14), Medtronic (0.12), and University of Bergen (0.1). Furthermore, we can see that the connections between the various agencies are very thin, indicating a lack of direct collaboration with each other.

### 3.3. Analysis of author

**Figure 5.**
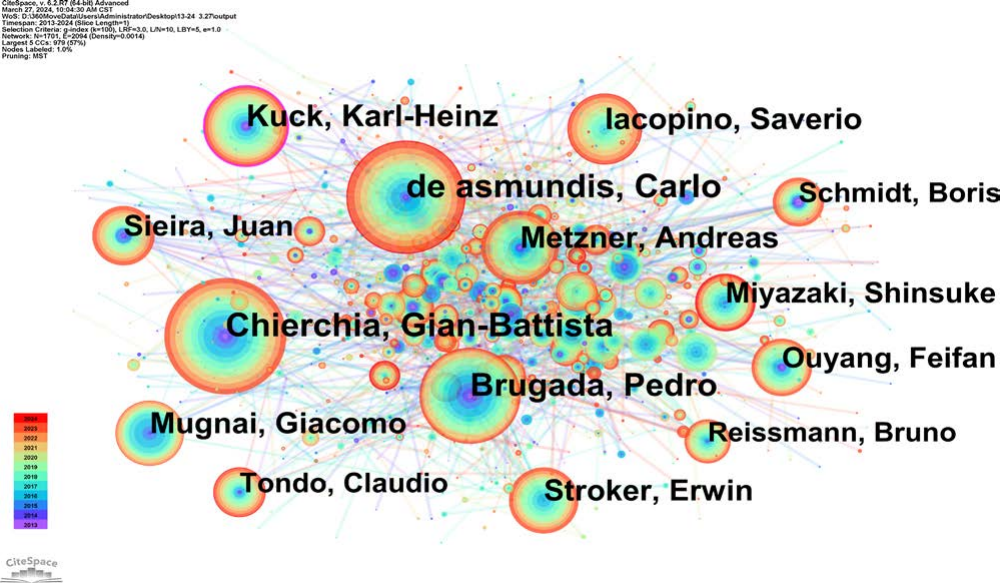
Author map researching atrial fibrillation and cryoballoon from 2013 to 2024.

8238 articles were published by 1701 authors. Experts in this discipline are the top 5 writers who have been written about (Table 2). There were 2094 linkages and 1701 nodes in the created map (Fig. 5).

**Table 2:** Top 5 authors in atrial fibrillation and cryoballoon in terms of centrality.

Ranking	Cited reference	centrality	Representative author (publication year)
1	121	0.11	Gian-Battista Chierchia (2013)
2	118	0.1	Carlo de Asmundis (2013)
3	100	0.1	Pedro Brugada (2013)
4	82	0.09	Karl-Heinz Kuck (2013)
5	77	0.07	Andreas Metzner (2013)

As the author with the most number of published works, G.-B. Chierchia is based at the Universitair Ziekenhuis Brussel in Belgium. Their team could provide clinical support to Pulsed electric field, cryoballoon, and radiofrequency for paroxysmal AF ablation^[13]^ (Table 2).

### 3.4. Analysis of co-cited references

**Figure 6.**
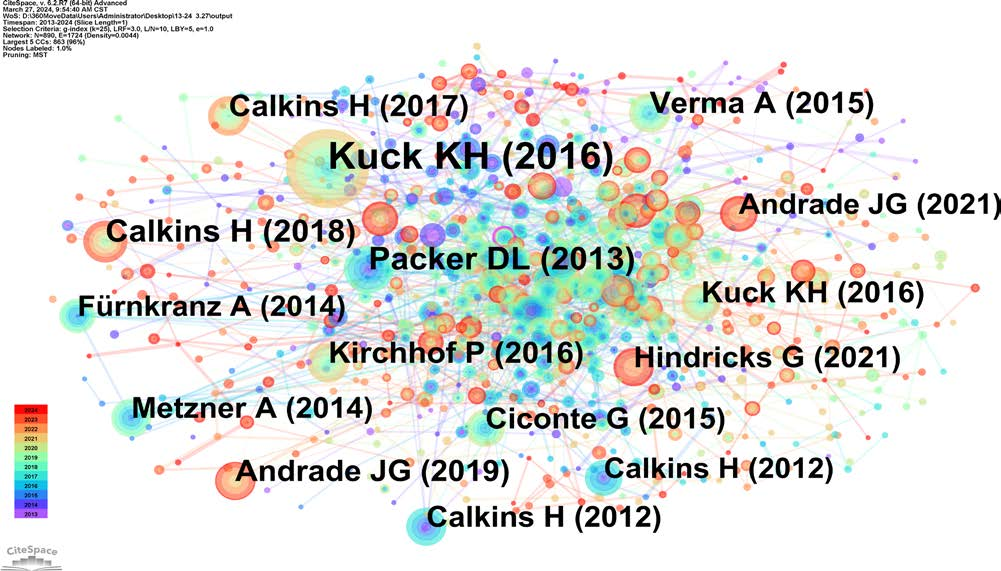
Co-cited references map researching atrial fibrillation and cryoballoon from 2013 to 2024.

It was discovered using a counts and centrality study (Figure 6, Table 3) that the data is typically presented as research articles. Of them, “Cryoballoon or Radiofrequency Ablation for Paroxysmal Atrial Fibrillation” was published in N Engl J Med in 2016. In this randomized trial, cryoballoon ablation was noninferior to radiofrequency ablation with respect to efficacy for the treatment of patients with drug-refractory paroxysmal AF, and there was no significant difference between the 2 methods with regard to overall safety. It can be seen that cryoballoon is getting more and more attention in AFs medicine.

**Table 3:** Top 5 co-cited references related to atrial fibrillation and cryoballoon research in terms of co-citation.

Ranking	Cited reference	Co-citation counts	Representative author (publication year)
1	Cryoballoon or radiofrequency ablation for paroxysmal atrial fibrillation	488	Kuck KH (2016)
2	Cryoballoon ablation of pulmonary veins for paroxysmal atrial fibrillation: first results of the North American Arctic Front (STOP AF) pivotal trial	193	Packer DL (2013)
3	2017 HRS/EHRA/ECAS/APHRS/SOLAECE expert consensus statement on catheter and surgical ablation of atrial fibrillation	163	Calkins H (2018
4	Approaches to catheter ablation for persistent atrial fibrillation	135	Verma A (2015)
5	Intra-articular injection of mesenchymal stem cells for the treatment of osteoarthritis of the knee: a Proof-of-Concept Clinical Trial	133	Calkins H (2017)

### 3.5. Analysis of keyword co-occurrence and burst analysis

**Figure 7.**
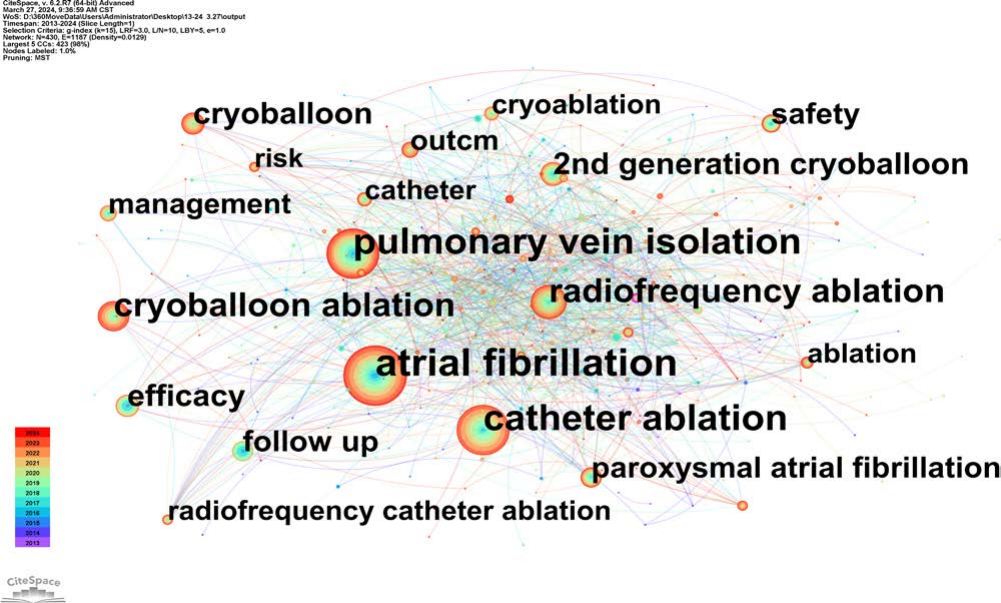
Co-occurrence keyword map researching atrial fibrillation and cryoballoon from 2013 to 2024.

A term co-occurrence map that was created contained 1187 linkages and 430 nodes (Fig. 7). A co-occurrence map of keywords could show popular subjects. It demonstrates that AF, radiofrequency ablation, catheter ablation, pulmonary vein isolation (PVI), and cryoballoon ablation were the most often used keywords.

**Figure 8.**
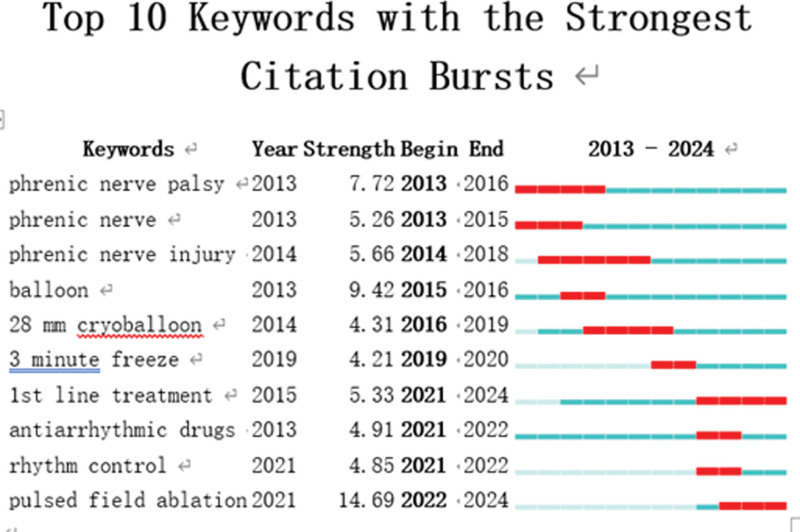
Top 10 keywords with the strongest citation bursts.

“Burst words” are words that are frequently used over long periods of time. It may be possible to predict the frontier of research trend by looking at the distribution of terms with the strongest citation explosion. The top 10 terms with the biggest increase in citations between 2013 and 2024 are shown in Figure 8. The keyword was cited occasionally, as indicated by the green bars, and frequently, as represented by the red bars. Terms like “phrenic nerve palsy,” “phrenic nerve injury,” “balloon,” “28 mm cryoballoon,” and “3 minute freeze” may be used a lot, suggesting that this field of study will attract more attention in the years to come.

## 4. Discussion

According to Citespace data, there were more yearly articles regarding cryoballoon and AFs between 2016 and 2023. It might be as a result of the growing emphasis that researchers are placing on cryoballoon, which is a crucial aspect of AFs. Probably because of the publication of “Cryoballoon or Radiofrequency Ablation for Paroxysmal Atrial Fibrillation,”^[16]^ it has been cited 593 times. It was discovered that, in terms of safety and effectiveness, PVI via cryoballoon ablation was not less effective than PVI via radiofrequency ablation when treating patients with drug-refractory paroxysmal AF. The main ablation technique used to treat individuals with paroxysmal AF is PVI.^[14,15]^ Acute PVI, however, does not ensure long-term electrical isolation of the pulmonary veins.^[15]^ Extended PVI has been made better by the use of more recent radiofrequency catheters equipped with contact-force sensing.^[15,17,18]^ Long-term PVI has also improved using the second-generation cryoballoon catheter.^[17,19]^ Beyond PVI, extensive wide-area circumferential ablation may provide additional ablation-related benefits, such as concurrent ganglionated plexus alteration.^[20]^ Nevertheless, the trial lacked sufficient power to evaluate the superiority of the first- or second-generation catheters. The ACC/AHA/ACCP improved its guidelines for AF diagnosis and treatment because of this article.^[21]^ We discovered that there are more publications on AFs cryoballoons in the United States than anywhere else. This could be because Chinese AFs research is less well-represented in English-language publications.

The Vrije Universiteit Brussel is the most influential in the field of cryoballoon, it includes some highly cited articles. i.e.,“Cryoballoon Ablation as Initial Treatment for Atrial Fibrillation: JACC State-of-the-Art Review” has been cited 30 times. “Early diagnosis and better rhythm management to improve outcomes in patients with AF: the 8th AFNET/EHRA consensus conference” has been cited 36 times. These highly cited articles not only make Vrije Universiteit Brussel the most published institution, but also have a very large influence in the field.

Analysis of keyword shows that the prevalent keywords were AF, catheter ablation, PVI, radiofrequency ablation, cryoballoon ablation. PVI is an established and effective treatment for drug-resistant AF.^[22]^ “Burst words” revealed that phrenic nerve palsy, phrenic nerve, phrenic nerve injury (PNI), balloon, 28-mm cryoballoon, 3-minute freeze may be used a lot, indicating that there will be more interest in this field of study in the years to come. PNP, or phenomenal nerve palsy, is the most common side effect seen after cryoballoon ablation. PNP incidence was reported to be greater following radiofrequency catheter ablation than during other procedures.^[16,23–26]^ Michifumi Tokuda found that While PNP was resolved after a year for the majority of patients following ablation, 0.6% of individuals experienced PNP during RSPVI that lasted longer than 4 years. When the right-sided pulmonary veins are ablationed, there is a markedly increased risk of PNI with cryoballoon ablation as opposed to radiofrequency ablation. The temperature at the time of PNI was found by Heeger et al^[27]^ to be a major predictor of PNI recovery. The likelihood of recovery within a year decreased with decreasing temperature at the time of PNI.

At 2016, the first study comparing the efficacy of a single “3-minute strategy” and the conventional “4-minute plus bonus freeze” application per vein following second-generation cryoballoon ablation for the treatment of PAF after 2 years follow-up. The main finding of this study was “single 3-minute strategy” showed equal efficacy as compared to the conventional 4-minute plus bonus freeze approach at 2-year follow-up, providing shorter procedure and fluoroscopy time. Nadir temperature and time to PVI were predictors of arrhythmic recurrences. “Bonus-freeze” strategy might be unnecessary.^[28,29]^

## 5. Conclusion

The USA, Germany, and Belgium have emerged as the 3 main research nations in this field with great publication rates and centrality. The strongest partnerships between developed countries and esteemed organizations are advantageous to the progress of cryoballoon and AF research. Because these articles have a high IF or serve as guidelines, they were frequently mentioned.

## Acknowledgments

The authors would like to express their appreciation to Professor Chaomei Chen for inventing Citespace and making it free to use.

## Author contributions

**Data curation:** Jing Lu, Nan Zhang, Dengfeng Ma.

**Formal analysis:** Jing Lu, Nan Zhang.

**Funding acquisition:** Jing Lu.

**Investigation:** Jing Lu.

**Software:** Jing Lu, Fan Yang, Yan Ren, Dengfeng Ma.

**Writing – original draft:** Jing Lu, Dengfeng Ma.

**Methodology:** Nan Zhang, Yu Gao.

**Project administration:** Fan Yang, Yu Gao.

**Resources:** Fan Yang, Yan Ren.

**Supervision:** Yan Ren, Dengfeng Ma.

**Conceptualization:** Dengfeng Ma.

**Visualization:** Dengfeng Ma.
